# Optimal Design Parameters of Thermal Flowmeter for Fuel Flow Measurement

**DOI:** 10.3390/s22228882

**Published:** 2022-11-17

**Authors:** Igor Korobiichuk, Andrii Ilchenko

**Affiliations:** 1 Łukasiewicz Research Network–Industrial Research Institute for Automation and Measurements PIAP, 02-486 Warsaw, Poland; 2 Department of Automobile and Transport Technologies, The State University “Zhytomyr Polytechnic”, 10005 Zhytomyr, Ukraine

**Keywords:** thermal flowmeter, flowmeter tube, heat transfer, fuel flow, design parameters

## Abstract

The article analyses the influence, relationship and value of the design parameters of the thermal flowmeter on its radial and axial heat fluxes in the tube. The purpose of the analyses is to check the change in the error of fuel flow measurement by the thermal flowmeter directly on the vehicle when using heating elements of different diameters. The influence of the radial heat flux of the flowmeter tube on the accuracy of fuel flow measurement is substantiated. Recommendations on the choice of design parameters of a thermal flowmeter at the stage of its design, development or use are developed under the condition of reducing the influence of the radial heat flow on the axial one, which will reduce the total error in the measurement of fuel flow rate.

## 1. Introduction

The consumption of liquid fuels in vehicles occupies the first place in the cost of transportation, so its accounting is not in doubt. This also applies to stationary power units, for example, for the production of electrical energy. Accounting is achieved by the development and implementation of devices, by means of which the consumption is measured. In vehicles, due to the specific operation, it is most preferable to use calorimetric flowmeters [1,2]. Nowadays there are descriptions of some flowmeter designs, where their technical characteristics are given [3,4,5]. However, the generalised analysis of design parameters and their influence on the process of measurement of fuel consumption of thermal flowmeters, suitable for operation on vehicles, is absent in scientific literature [6,7,8,9,10]. Separate information about some thermal flowmeters exists, but in general it can be argued that systematised information on the use of thermal flowmeter to determine fuel consumption in vehicles is just beginning to appear [11,12,13]. For use in vehicles, one of the most promising is a flowmeter [14,15].

Ren, R. [16] studied a high-precision ultrasonic flowmeter based on the cross-correlation method is designed, and the commercial finite element software COMSOL Multiphysics 5.6 is used to simulate the propagation process of ultrasonic waves. The results can be used for high flow velocity (26 m/s) and a pipe diameter in the range of DN6~DN1600. Hu, Y.-C [17] studied at simulating the fluid–structure coupling dynamics of a dual U-tube Coriolis mass flowmeter through the COMSOL simulation package. However, the zero drift will occur when the dual U-tube structure is unbalanced that can be appeared on the board of car. Sutardi [18] analysed a numerical study of square edge and quadrant edge orifice flow meter performance with different diameter ratios and Reynolds numbers for orifice plate flow meter. de Oliveira [19] numerical simulations were performed in Computational Fluid Dynamics based on the finite volume technique in three dimensions, using a non-homogeneous model, through the ANSYS Fluent software, for a Venturi meter in vertical and horizontal positions. The possibility of evaluating the efficiency of the heat exchanger element with a special plate stamping is presented [20], which is based on the results of computer simulation. Hafeez, M.B. [21] probed the creation of heat energy and concentrated it into Newtonian liquids across vertical 3D-heated plates. The study found that the generation of thermal energy for hybridised nanomaterials is much higher. 

Based on previous studies and analyses, results have been obtained that can be used in the design of heat exchange elements of new structures with optimal parameters for high-efficiency heating of liquid heat transfer fluids [16,17,18,19,20,21].

The purpose of this study is to analyse the influence of design parameters of a thermal flowmeter (tube and heater diameters, tube material) on heat flow, as well as the development of recommendations for their choice in terms of reducing the error in measuring the consumption of petroleum and mixed fuels for internal combustion engines.

## 2. Materials and Methods

The measurement of the parameters for liquid substances is widely used in many sectors of the economy: engineering, oil production and refining, agriculture, transport, etc. The parameter of liquid flow is the amount of substance flowing through the cross-section of the pipeline in a unit of time and the total amount of transported substance (total flow rate).

**Figure 1:**
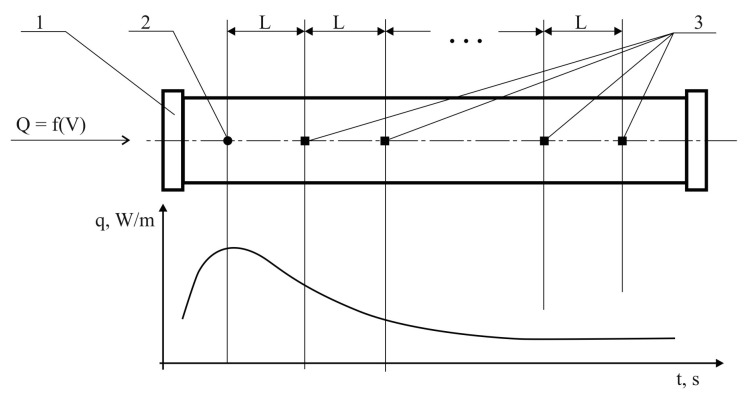
Heat transfer diagram in the axial direction of the thermal flowmeter tube: 1, tube; 2, heater; 3, thermal sensors; Q, fuel flow, l/s; V, fuel flow rate, m/s; L, distance between thermal sensors, m; q, heat flow, W/m.

Heat transfer in the axial direction is analysed in thermal flowmeter tubes (calorimeters and thermo anemometers) (Figure 1). However, this transfer exists in both the axial and radial directions. At the same time, it is interrelated and mutually influential. To determine the parameters of fuel movement, the temperature distribution along the flowmeter tube axis is analysed [22,23,24].

Radial heat flow can be determined [24]:

(1) $$q_{1}=\frac{2\pi }{ln\left(\frac{d_{2}}{d_{1}}\right)}\times \frac{\lambda_{2}\lambda_{1}}{\lambda_{2}+\lambda_{1}}\times \left(t_{1}-t_{2}\right),$$

where *d*_1_–heater diameter, m; *d*_2_–outer diameter of the flowmeter tube, m; *t*_1_–heater temperature, °K; *t*_2_–temperature of the outer surface of the flowmeter tube (ambient temperature in the absence of thermal insulation of the tube), °K; *λ*_1_, *λ*_2_–thermal conductivity of fuel and flowmeter tube material, W/(m∙K).

The diameter of the pipe increases the distance from the heater to this surface, as well as the area of the outer surface of the pipe, which will lead to its greater cooling, that is, it will affect the “external surface-heater” temperature difference, which will change the heat flux. An increase in the diameter of the heater will also lead to a decrease in the specified distance and, as a result, an increase in the temperature of the outer surface of the pipe, which will also change the heat flux. 

As the pipe diameter increases, the radial heat flux will decrease. The article discusses the conditions under which it is possible to avoid the radial flow and, accordingly, its influence on the axial one.

The first fraction (1) includes only the design parameters of the flowmeter tube (outer diameter *d*_2_ and heater diameter *d*_1_), so it is called the design factor of the thermal flowmeter tube [24]:

(2) $$K_{K}=\frac{2\pi }{\ln\left(\frac{d_{2}}{d_{1}}\right)}$$

From (2), it can be established that the value of the radial heat flow *q*_1_ will be affected only by the thermal conductivity of the fuel and the material of the flowmeter tube, provided that

(3) $$\ln\left(\frac{d_{2}}{d_{1}}\right)=2\pi$$

or the ratio of the outer diameter of the flowmeter tube to the diameter of the heater is

(4) $$\frac{d_{2}}{d_{1}}=e^{2\pi }\approx 534$$

From (4), it is easy to find that the radial heat flux of a thermal flowmeter with a cylindrical wall (linear heat flux density) does not depend on the wall thickness of the flowmeter tube. It significantly decreases as the design factor of the flowmeter tube approaches unity, i.e., as the ratio of the outer diameter of the flowmeter tube to the diameter of the heater increases. Thus, to reduce the influence of radial heat flux in the thermal flowmeter on the axial heat flux (reducing the accuracy of fuel flow measurement), it is necessary to reduce the heater diameter and increase the diameter of the thermal flowmeter tube. Moreover, the ratio of diameters in (4) is not desirable to choose a value greater than 534, as its further growth will lead to values of the design factor less than unity and then again to an increase in measurement error.

For design reasons, ensuring that the ratio of the thermal flowmeter tube diameter to the heater diameter is 534 does not seem convenient for use in transportation. Alternatively, the thinnest possible wire heater can be used along the axis of the flowmeter tube.

The second fraction in (1) characterizes the radial thermal conductivity of the flowmeter tube and is called the radial thermal conductivity coefficient of the thermal flowmeter tube [24]:

(5) $$K_{PT}=\frac{\lambda_{2}\lambda_{1}}{\lambda_{2}+\lambda_{1}}$$

Its value is influenced more by the thermal conductivity of the thermal flowmeter tube material than by the thermal conductivity of the fuels, since their value may differ depending on the tube material by several orders of magnitude (Table 1).

Table 1 shows that the average thermal conductivity of the fuel is 0.11 W/(m∙K). If ebonite (0.16 W/(m∙K)) is used as a material for the flowmeter tube, the radial thermal conductivity coefficient of the heat meter tube is 0.0652. If a tube with relatively high thermal conductivity is used and a material such as Monel (14.9 W/(m∙K)) is selected, the radial thermal conductivity coefficient of the heat meter tube increases to 0.1092. An alloy of metals 95% Al + 3–5% Cu + 0.5% Mg as the material of the heat flowmeter tube with even higher thermal conductivity (181 W/(m∙K)) will already give a slight increase in the coefficient of radial thermal conductivity of the heat flowmeter tube to 0.1099.

In order to completely exclude the influence of thermal conductivity of the flowmeter tube material on radial heat flow in (1), i.e., on the measurement error of oil fuel flow rates, it is necessary to find from (5) such a value of thermal conductivity coefficient of tube material, at which its radial thermal conductivity coefficient is equal to one. Calculations show that it is possible only theoretically because such materials do not exist in nature. That is, complete elimination of radial heat transfer in the tube of a thermal flowmeter by the choice of the tube material is impossible. Including the use of external thermal insulation of the flowmeter tube cannot reduce the radial heat flow to zero, because there will always be a temperature difference between the heater and the outer surface of the tube of the flowmeter.

To exclude the joint influence of the design factor and the radial thermal conductivity coefficient of the heat meter tube on the radial heat flow when measuring the consumption of petroleum fuels, we can try to equating their product to unity, considering (4):

(6) $$\frac{2\pi }{\ln\frac{d_{2}}{d_{1}}}\frac{\lambda_{2}\lambda_{1}}{\lambda_{2}+\lambda_{1}}=1$$

From (6), it is possible to establish a better ratio of the diameter of the thermal flowmeter tube to the diameter of the heater, provided that their joint influence and the influence of the thermal conductivity of the tube on the radial heat flow (flow measurement error) of petroleum fuels moving in the flowmeter tube is minimal. The results of these calculations for some materials of the thermal flowmeter tube are given in Table 2.

## 3. Results and Discussion

**Figure 2:**
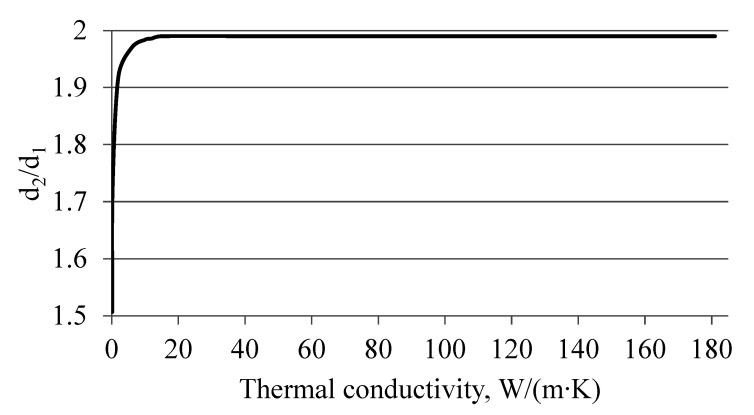
Dependence of *d*_2_/*d*_1_ (ratio of the outer diameter of the flowmeter tube to the diameter of the heater) on the thermal conductivity of various materials, subject to the slightest influence on the radial heat flow.

The dependence of the geometric parameters of the thermal flowmeter tube on the tube material under the condition of minimal influence on the radial heat flux (error in determining the fuel flow rate) is shown in Figure 2.

As shown above, the radial heat flux in the thermal flowmeter tube depends on the product of the design factor, the radial thermal conductivity coefficient of the tube and the temperature difference between the heater and the outer surface of the flowmeter tube. To reduce the influence of the radial heat flux on the axial heat flux (and thus on the accuracy of fuel flow measurement of internal combustion engines), it is necessary to choose such design parameters (material and diameter of the thermal flowmeter tube, heater diameter) so that the ratio of these diameters corresponds to the material of the thermal flowmeter tube.

Analysis of Figure 2 shows that the ratio d_2_/d_1_ for materials with a thermal conductivity coefficient of 0.16 … 12 W/(m∙K) should be in the range of 0.507 … 1.984. This will reduce the influence of radial heat flux on the axial one and thus reduce the fuel flow measurement error. Depending on the values of thermal conductivity of the thermal flowmeter tube material, they should change according to the dependencies (Table 3).

**Figure 3:**
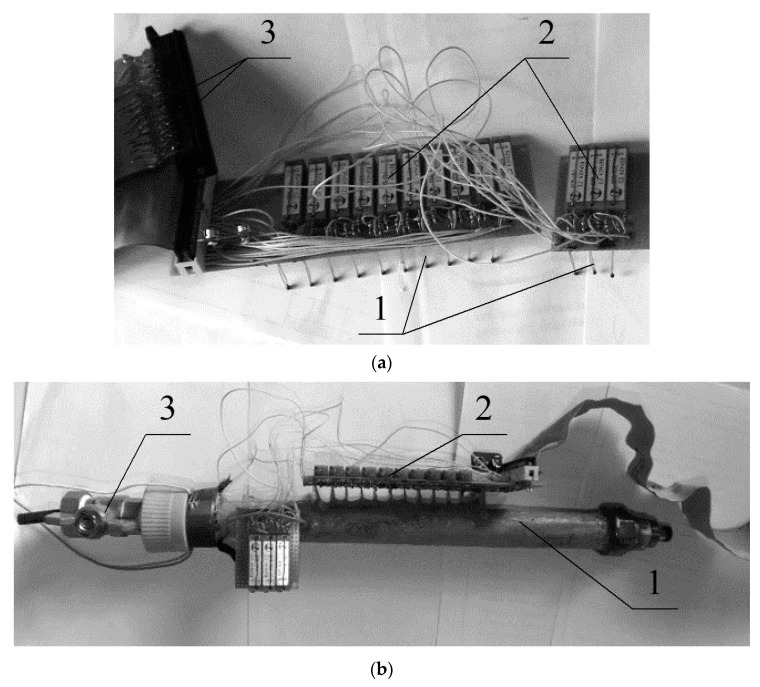
Thermal flowmeter: (**a**) board with measuring bridges (1, thermal converters; 2, balancing resistors of measuring bridges; 3, connector), (**b**) flowmeter prototype a (1, flowmeter tube; 2, board with balancing resistors and thermal converters; 3, fuel tap through the flowmeter).

**Figure 4:**
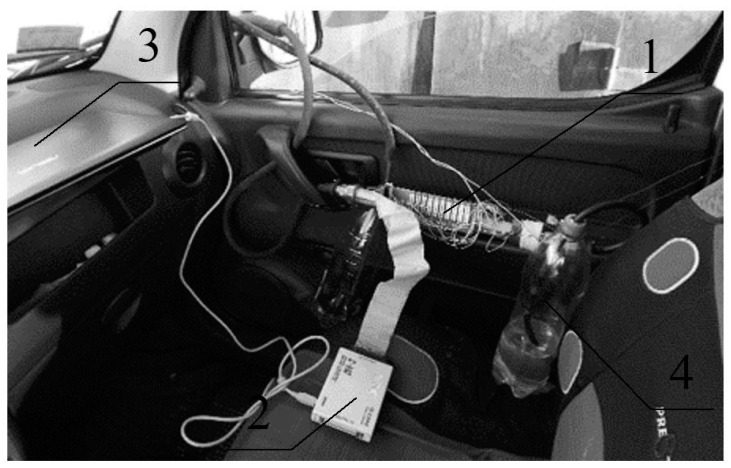
Carrying out experimental studies on the vehicle: 1, flowmeter; 2, analog–digital converter; 3, computer; 4, vessel with fuel.

To check the influence of the diameter of the heating element of the thermal flowmeter on the accuracy of fuel flow measurement, a prototype of the thermal flowmeter was designed and made (Figure 3). The flowmeter was installed in the fuel line of the vehicle (Figure 4), the fuel consumption was then measured.

The power supply of the heating element and measuring bridges was used as a power supply of B5-47 with an output voltage of 12 V. Measuring bridges were balanced at 20 °C.

Preliminary calibration of measuring bridges was carried out [24,25]. Preliminary calibration of measuring bridges for each of the channels in the temperature range of 20 … 96 °C was carried out and the voltage dependence on the temperature was obtained every 2 °C.

**Figure 5:**
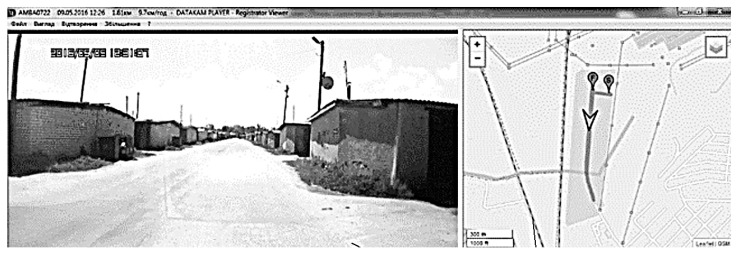
Example of the RegistratorViewer software package operation for traffic route.

**Figure 6:**
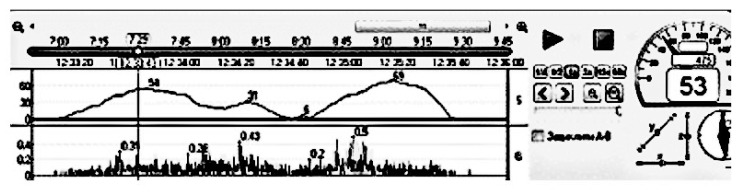
Example of registering fuel consumption and vehicle speed over time in the process of setting up the RegistratorViewer software complex.

The data from the flowmeter were fed to a personal computer through an analog–digital converter and processed by the RegistratorViewer software package (Figure 5). The example of setting up the complex is shown in Figure 6, which shows the change in vehicle speed over time and fuel consumption (in relative units). When processing the results of measurements at constant vehicle speeds, the fuel consumption was reduced to the dimension of l/100 km. Performance testing of the developed system was performed and also configured for various transient speeds (Figure 6). The fuel flow rate was also measured in parallel with the volumetric method. 

In the first case, a nichrome wire with high electric resistance (X20H80 nichrome) in the form of a cylindrical coil with a diameter of 10 mm was used as a heating element. In the second case, the nichrome heating element was coaxial with the flowmeter tube. Figure 5 shows an example of the program and confirmation of the experiment. As a result of the experiment, it was found that with decreasing the diameter of the heating element, the accuracy of fuel flow measurement decreased by 1.2 … 1.6 times.

Experimental studies were conducted to check the influence of the diameter of the heating element on the measurement error of the fuel consumption. Experimental studies have confirmed the influence of the diameter of the heating element on the error of fuel consumption measurement. During the experimental studies, two types of heating elements were installed in the thermal flowmeter tube made of nichrome X20H80: in the first case, a straight wire with a length of 38 mm was located along the axis of the flowmeter tube, in the second case, a wire of the same length had the shape of a spiral (one turn with a diameter of 12 mm), and the axis of the spiral circle was located along the axis of the flow meter tube. Measurements were carried out (Figure 4 and Figure 5) on a straight horizontal section of the road with an asphalt surface at a speed of 50 km/h, the driving distance was 1.2 km (Table 4). Fuel consumption was determined by the volumetric method and with the help of a thermal flowmeter with installed heating elements of various shapes.

The obtained experimental studies showed that reducing the diameter of the wire heating element from 12 mm to the minimum possible (to the diameter of the wire when it is installed axially along the axis of the tube of the heat flow meter) with an unchanged diameter of the tube allows to reduce the relative error of fuel consumption by 1.6 times.

The result of the study is that the diameter of the heating element installed in the tube of the heat flow meter affects the measurement error of the fuel consumption, and to reduce this error, it is necessary to reduce it. The obtained results make it possible to develop recommendations for the selection of design parameters of the flow meter tube to increase measurement accuracy.

## 4. Conclusions

1. To reduce the error of fuel flow measurement due to reducing the influence of the radial heat flux in the thermal flowmeter on the axial heat flux (without taking into account the thermal conductivity of fuel and the material of the flowmeter tube), it is necessary to reduce the diameter of the heater and increase the diameter of the thermal flowmeter tube.
2. The dependences of the ratio of the tube diameter to the diameter of the heater of the thermal flowmeter for the tube materials of different thermal conductivity are obtained, allowing to reduce the influence of the radial heat flux on the axial, which will eventually reduce the error of measuring the fuel flow.
3. It was found experimentally that the diameter of the heating element in the tube of the thermal flowmeter affects the error of fuel flow measurement, and its reduction allows to reduce this error. It was experimentally established that reducing the diameter of the wire heating element from 12 mm to the minimum possible diameter (to the diameter of the wire when it is installed axially along the axis of the tube of the heat flow meter) allows to reduce the relative error of fuel consumption by 1.6 times, with other unchanged design parameters of the tube.

## Figures and Tables

**Table 1:** Thermal conductivity of petroleum fuels and different flowmeter tube materials, *t* = 20 °C [9,10].

No.	Material/Substance	Thermal Conductivity, W/(m∙K)
Fuel		
1	Gasoline	0.106
2	Diesel fuel	0.108
3	Kerosene	0.109
4	Crude oil	0.114
Metal		
1	Monel: Cu 12% + Fe 25% + others 63%	14.9
2	Brass	110
3	Duralumin	160
4	95% Al + 3–5% Cu + 0.5% Mg	181
Others		
1	Fiberglass	0.036
2	Lightweight/heavyweight foam glass	0.06/0.08
3	Mipor	0.085
4	Rubber	0.15
5	Ebonite	0.16
6	Fluoroplastic F-5	0.25
7	Glass fiber board	0.3

**Table 2:** Selection of geometric parameters and material of the thermal flowmeter tube.

Flowmeter Tube Material	Thermal Conductivity, W/(m∙K)	*d*_2_/*d*_1_
Ebonite	0.16	1.507
Fluoroplastic F-5	0.25	1.62
Hypothetical materials (with acceptable thermal conductivity)	2	1.92
	6	1.97
	10	1.98
	12	1.984
Monel	14.9	1.99
Hypothetical materials (with acceptable thermal conductivity)	50	1.99
	100	1.99
95% Аl + 3–5% Cu + 0.5% Mg	181	1.99
Hypothetical materials (with acceptable thermal conductivity)	250	1.99
	300	1.99

**Table 3:** Dependencies of d_2_/d_1_ change on thermal conductivity of the thermal flowmeter tube material (provided that the influence of its radial heat flux on the axial heat flux is reduced).

Thermal Conductivity Coefficient, W/(m∙K)	Dependencies (Value)	Reliability of Approximation, R^2^
0.16 … 6	*d*_2_/*d*_1_ = 0.1182 ln(*λ*_2_) + 1.7959	0.9
6 … 12	*d*_2_/*d*_1_ = 0.0028 *λ*_2_ + 1.954	0.95
>12	*d*_2_/*d*_1_ = 1.99	-

**Table 4:** Determination of fuel consumption by volumetric method and using a thermal flowmeter with heating elements of different shapes.

The Number of the Experiment	Fuel Consumption, Which Was Measured by the Volumetric Method, l/100 km (Option 1)	Fuel Consumption, Which Was Measured with the Heating Element Located along the Axis of the Flow Meter Tube, l/100 km (Option 2)	Fuel Consumption, Which Was Measured with a Heating Element Located along the Axis of the Tube in the Form of a Spiral, l/100 km (Option 3)	Relative Error between Options 1 and 2, %	Relative Error between Options 1 and 3, %
1	6.12	6.363	6.531	3.84	6.29
2	6.111	6.37	6.512	4.07	6.16
3	6.123	6.343	6.466	3.47	5.3
4	6.142	6.331	6.467	2.99	5.03
5	6.117	6.354	6.468	3.73	5.43
6	6.129	6.355	6.477	3.56	5.37
7	6.151	6.274	6.499	1.96	5.35
8	6.139	6.367	6.486	3.58	5.35
9	6.121	6.372	6.469	3.94	5.38
10	6.145	6.376	6.564	3.62	6.38
Average value	6.13	6.35	6.49	3.47	5.6

## Data Availability

Not applicable.
